# Microbial imbalance and intestinal pathologies: connections and contributions

**DOI:** 10.1242/dmm.016428

**Published:** 2014-10

**Authors:** Ye Yang, Christian Jobin

**Affiliations:** 1 Department of Medicine, University of Florida, Gainesville, FL 32611, USA.; 2 Department of Infectious Diseases and Pathology, University of Florida, Gainesville, FL 32611, USA.

**Keywords:** Adherent-invasive *E. coli*, Dysbiosis, IBD, CRC, Colibactin

## Abstract

Microbiome analysis has identified a state of microbial imbalance (dysbiosis) in patients with chronic intestinal inflammation and colorectal cancer. The bacterial phylum Proteobacteria is often overrepresented in these individuals, with *Escherichia coli* being the most prevalent species. It is clear that a complex interplay between the host, bacteria and bacterial genes is implicated in the development of these intestinal diseases. Understanding the basic elements of these interactions could have important implications for disease detection and management. Recent studies have revealed that *E. coli* utilizes a complex arsenal of virulence factors to colonize and persist in the intestine. Some of these virulence factors, such as the genotoxin colibactin, were found to promote colorectal cancer in experimental models. In this Review, we summarize key features of the dysbiotic states associated with chronic intestinal inflammation and colorectal cancer, and discuss how the dysregulated interplay between host and bacteria could favor the emergence of *E. coli* with pathological traits implicated in these pathologies.

## Introduction

Inflammatory bowel disease (IBD) is a group of inflammatory disorders of the intestine. The most common forms of IBD are ulcerative colitis (colitis of the large intestine) and Crohn’s disease (a type of IBD that can affect any part of the digestive tract), which combine to affect ~1.4 million Americans (~0.45% of the US population), with up to 70,000 new cases being diagnosed in the United States each year (CCFA, 2011). IBD is a multifactorial immune disorder: it is influenced by the genetic susceptibility of an individual and by environmental and lifestyle factors (diet, smoking, etc.) (Kaser et al., 2010). Although IBD predominantly affects people in the Western world (e.g. Europe and North America), both the incidence and the prevalence of this pathology have risen in Asia, especially in areas that have adopted an industrialized lifestyle (Ng et al., 2012; Prideaux et al., 2012). Current treatment for IBD, which mainly involves anti-inflammatory drugs (e.g. mesalazine), corticosteroids (e.g. prednisone), immunosuppressant drugs (e.g. azathioprine), biological therapy [e.g. tumor necrosis factor α (TNF-α) inhibitors such as infliximab and adalimumab] and surgery, is costly and often associated with severe adverse effects (Baumgart and Sandborn, 2012; Ordás et al., 2012). IBD is associated with severe morbidity and impaired quality of life owing to its chronic nature and high recurrence (Høivik et al., 2013; Netjes and Rijken, 2013), and represents a substantial socioeconomic burden in the United States (~$2.2 billion/year, including healthcare costs and loss of earnings) (CCFA, 2011).

Colorectal cancer (CRC) is the type of cancer that develops in the colon or rectum. Despite the increasing implementation of colonoscopy screening and advances in chemotherapeutic and biological-agent-based therapies, CRC remains one of the most common and deadliest malignancies in the United States (Siegel et al., 2014). The American Cancer Society estimates that, in 2014, there will be ~140,000 new CRC cases and over 50,000 CRC-related deaths nationwide (Siegel et al., 2014). CRC includes hereditary, sporadic and colitis-associated CRC. Colitis-associated CRC represents a severe medical complication for patients with IBD, and often shows rapid progression, poor response to treatment and high mortality (Feagins et al., 2009). Individuals with IBD are at increased risk for developing CRC (Herrinton et al., 2012).

Both IBD and CRC evolve in the context of a vast and complex gut microbial ecosystem. The human intestinal microbiota consists mainly of bacteria (~10^14^) and contains more than 10^6^ bacterial genes (Human Microbiome Project Consortium, 2012). Through the action of various microbial structural components, microbial gene products and/or metabolites, this microbiota plays essential roles in intestinal homeostasis, regulating host immunity, gut barrier function and metabolic activity (Clemente et al., 2012). Changes in the richness, diversity and stability of the gut bacterial ecosystem, a state referred to as microbial dysbiosis, is commonly associated with intestinal pathologies such as IBD and CRC (discussed below). In this Review, we will summarize key characteristics of the microbial dysbiosis associated with these intestinal diseases, and discuss connections between the gut microbiota, intestinal inflammation and carcinogenesis. We will focus on potential microbial candidates – specifically *Escherichia coli* – that have been identified in studies of the human CRC microbiota and are implicated in the carcinogenesis. We will describe important biological events implicated in the development of microbial dysbiosis and the emergence of carcinogenic microorganisms. Finally, we will review recent studies that demonstrate the pro-tumorigenic role of *E. coli* in animal models, in particular of those strains that produce the genotoxin colibactin.

## Microbiota and intestinal pathologies

In the 1990s, it was found that re-routing the intestine out onto the surface of the skin to divert the fecal stream away from a patient’s bowel prevented recurrence of Crohn’s disease (Rutgeerts et al., 1991). Fecal-stream diversion also induced clinical and histopathological remission of collagenous colitis, a type of IBD that specifically affects the colon (Järnerot et al., 1995). These findings suggest that some agents that are present in the luminal contents are important for IBD pathogenesis. Consistent with this concept, reinfusion of the luminal contents into bypassed colonic segments caused recurrent Crohn’s disease (D’Haens et al., 1998). Later, studies using genetically engineered animal models of IBD showed that colitis was often attenuated or absent when these animals were kept in germ-free conditions (Kamada et al., 2013), indicating that the gut microbiota is essential for triggering and/or enhancing chronic intestinal inflammation. This is supported by the observation that antibiotic treatment ameliorates clinical symptoms in certain IBD patients (Feller et al., 2010; Selby et al., 2007; Thia et al., 2009). Moreover, transplantation of the microbiota from genetically engineered IBD mice to healthy wild-type mice induced colitis in the recipients (Grivennikov, 2013), although similar experiments have not been performed with the human dysbiotic microbiota. Nevertheless, an accumulating body of evidence supports the idea that intestinal microbes play important roles in the etiology and pathology of IBD.

More recently, researchers have started to unravel the genetic factors that contribute to IBD pathogenesis. The first polymorphism associated with susceptibility to Crohn’s disease was identified in the innate immunity sensor gene *NOD2* (nucleotide-binding oligomerization domain-containing protein 2) in 2001 (Hugot et al., 2001; Ogura et al., 2001). Thus far, more than 160 genomic loci have been correlated with IBD susceptibility in humans (Jostins et al., 2012). Noticeably, many of these IBD genetic loci are implicated in innate and adaptive immunity, gut barrier function, and bacterial handling (Jostins et al., 2012). However, the low concordance rates of IBD between monozygotic twins indicate that genetic predisposition only partially accounts for susceptibility to this disease (Halme et al., 2006). Moreover, evidence that IBD susceptibility genes promote microbial imbalance in the intestine is lacking. These observations clearly highlight the complexity of this intestinal pathology; as such, determining the events that lead to IBD development will require a holistic view and an integrative approach to understanding host-bacteria interactions.

In the last decade, advancements in DNA sequencing technologies and sequence analysis have enabled a comprehensive characterization of the gut microbiota at an unprecedented level. Many studies have shown that the intestinal microbial ecosystem is markedly altered in individuals with IBD as compared to healthy individuals (Manichanh et al., 2012). These microbial composition analyses, combined with functional studies, have revealed dynamic roles of the microbiota in IBD. The pathology is likely caused by the action of various groups of bacteria (polymicrobial), either through the production of noxious factors and/or antigens or through the depletion of protective mediators such as short-chain fatty acids (SCFA) (Kamada et al., 2013; Kostic et al., 2014; Strober, 2013). The dual nature of the microbiota, pathogenic or protective, highlights the complex relationship that exists between microbes and their host (Kamada et al., 2013).

To date, several enteropathogens (e.g. *Mycobacterium avium* subspecies *paratuberculosis*, *Yersinia* spp., *Listeria monocytogenes*, *Salmonella* spp., *Campylobacter concisus*) that are found to be abundantly present in some IBD cases have been speculated to contribute to the disease pathogenesis (Chassaing and Darfeuille-Michaud, 2011; Mukhopadhya et al., 2012). However, there has been no evidence that these pathogenic microorganisms cause IBD (Packey and Sartor, 2009). By contrast, some bacteria [e.g. *Faecalibacterium prausnitzii* (Sokol et al., 2008), *Clostridium* spp. (clusters IV and XIVa) (Atarashi et al., 2011), commensal *Bacteroides fragilis* (Round et al., 2011)] that are able to induce immunosuppressive responses are considered to be protective against IBD. Bacteria can also enhance gut epithelial barrier function by stimulating mucus secretion and producing certain metabolites. For example, *Bifidobacterium* spp. protect mice from the lethal action of enterohemorrhagic *E. coli* O157:H7 infection through the enhanced production of acetate, an effect linked to the inhibition of translocation of shiga toxin (a toxin produced by this *E. coli* strain) from the lumen to the gut tissue (Fukuda et al., 2011; Fukuda et al., 2012). For more information on the role of bacteria in modulating intestinal homeostasis and inflammation, we direct the reader to a series of recent Reviews (Hardy et al., 2013; Kamada et al., 2013; Kostic et al., 2014; Strober, 2013).

Cancer genome studies have linked many somatic mutations to CRC (Esteban-Jurado et al., 2014; Watson et al., 2013). But, similarly to IBD, the etiology of CRC can only be partially attributed to genetics (Rustgi, 2007), indicating the involvement of environmental factors in this disease pathogenesis. Noticeably, marked changes in the intestinal microbiota are also observed in individuals with CRC (Ahn et al., 2013; Castellarin et al., 2012; Chen et al., 2012; Kostic et al., 2012; McCoy et al., 2013; Sobhani et al., 2011; Wu et al., 2013). Accumulating evidence suggests that the microbiota influences intestinal carcinogenesis. In particular, a number of studies using germ-free animals have revealed that the microbiota has cancer-promoting effects in spontaneous and genetically induced cancer models (Schwabe and Jobin, 2013; Sears and Garrett, 2014).

The microbiota might promote colonic carcinogenesis via a variety of mechanisms, and we direct readers to several recent reviews for more information on this topic (Elinav et al., 2013; Grivennikov, 2013; Schwabe and Jobin, 2013). Some key mechanisms include the pro-tumorigenic inflammatory responses induced by the dysbiotic microbiota, the inflammation-associated tissue injury and repair process, and the expansion of ‘keystone’ bacteria – those harboring specific virulence traits to drive colon cancer development. These ‘alpha bugs’, which usually exist at very low levels at gut homeostasis, could contribute substantially to colonic carcinogenesis when overrepresented (Sears and Pardoll, 2011). Thus far, several ‘alpha bugs’ that promote intestinal carcinogenesis in animal models have been described: enterotoxigenic *B. fragilis* (Wu et al., 2009), *Fusobacterium nucleatum* (Kostic et al., 2013; Rubinstein et al., 2013) and colibactin-producing *E. coli* (Arthur et al., 2012; Bonnet et al., 2014; Cougnoux et al., 2014).

It is worth noting that the microbiota might also prevent carcinogenesis. Recent work by Zhan et al. demonstrates that the gut microbiota protects mice from intestinal carcinogenesis induced by epithelial injury (Zhan et al., 2013). The authors attributed the beneficial effects of the microbiota to its ability to promote epithelial restitution and injury recovery. Key mechanisms by which commensal bacteria suppress colonic carcinogenesis include the induction of immunosuppressive responses, the detoxification of carcinogens and the production of cancer-suppressing metabolites (Boleij and Tjalsma, 2012; Schwabe and Jobin, 2013).

## Dysbiotic states during inflammation and carcinogenesis

Although a considerable degree of variation is found among the microbial compositions of different individuals, the gut microbiota of healthy adults is commonly dominated by four major bacterial phyla: Firmicutes and Bacteroidetes (two groups of obligate anaerobes), which constitute ~90% of the microbial ecosystem, and Proteobacteria and Actinobacteria, which contribute to a lesser degree (Human Microbiome Project Consortium, 2012).

The structure of the human gut microbiota is established early in life and remains relatively stable for decades (Costello et al., 2009; Yatsunenko et al., 2012). However, during chronic intestinal inflammation, as experienced by individuals with IBD, the gut microbial composition displays marked alterations both taxonomically and functionally (Kostic et al., 2014; Manichanh et al., 2012). In general, the IBD-associated gut microbiota exhibits lower stability and diversity as compared with the microbial community found in a healthy human gut. At the phylum level, levels of both Firmicutes and Bacteroidetes are decreased, whereas those of Proteobacteria and Actinobacteria are significantly increased. Concurrently, there is a marked drop in the abundance of protective anaerobic commensals, mostly Firmicutes (e.g. *F. prausnitzii, Clostridium* spp.) and Bacteroidetes (e.g. *B. fragilis*), and an expansion of translocating facultative aerobes, especially bacteria belonging to the Enterobacteriaceae family (phylum Proteobacteria). Overall, the gut microbiota of individuals with IBD has a higher prevalence of Gram-negative bacteria, largely owing to the reduced ratio of Firmicutes (Gram-positive) relative to Bacteroidetes (Gram-negative).

In addition to the structural imbalance, profound perturbations of the functions of gut microbiota are also observed in IBD, e.g. bacterial amino acid biosynthesis and carbohydrate metabolism are diminished, whereas nutrient uptake is enhanced (Morgan et al., 2012). Importantly, bacterial genes involved in survival and pathogenesis processes, such as redox tolerance, secretion systems, adherence and/or invasion, are overrepresented in the microbiota of individuals with ileal Crohn’s disease, indicative of a functional shift to a ‘pathogenic’ community (Morgan et al., 2012). Conversely, pathways linked to the production of bacterial SCFAs, microbial metabolites known for their immunosuppressive functions (Arpaia et al., 2013; Furusawa et al., 2013; Maslowski et al., 2009; Singh et al., 2014; Smith et al., 2013), are repressed (Morgan et al., 2012). Using the *T-bet*^−/−^×*Rag2*^−/−^ ulcerative colitis (TRUC) mouse, an animal model of ulcerative colitis that features expanded Enterobacteriaceae (Garrett et al., 2010), Rooks et al. recently reported increased bacterial motility, tetrathionate respiration [a metabolic pathway promoting intestinal colonization of the pathogenic *Salmonella enterica* subsp. Typhimurium in the inflamed gut (Winter et al., 2010)], and benzoate degradation [a pathway linking to *Enterobacteriaceae* growth and virulence (Freestone et al., 2007; Lyte et al., 2011)] in active colitis (Rooks et al., 2014). It is not clear whether changes in microbial activities influence IBD pathogenesis. However, it is reasonable to speculate that improper microbial function would impact mucosal immune responses (Kamada et al., 2013; Kostic et al., 2014; Strober, 2013). Interestingly, therapeutic interventions, such as anti-TNF-α therapy, in TRUC mice impacts gut microbiota composition (Rooks et al., 2014), revealing the plasticity of the microbiota to external cues.

*E. coli*, a key member of the Enterobacteriaceae family, is a common colonizer of the human intestine, with an average of five commensal *E. coli* strains found in a human digestive tract (Apperloo-Renkema et al., 1990). In healthy individuals, Enterobacteriaceae constitute only a small fraction (less than 1%) of the gut microbiota (Eckburg et al., 2005). However, Enterobacteriaceae, in particular *E. coli*, become dominant in the gut microbiota of individuals with IBD and in several animal models of gut inflammation (Carvalho et al., 2012; Morgan et al., 2012; Mukhopadhya et al., 2012). *E. coli* strains isolated from individuals with IBD are often adherent and invasive, displaying pathogenic properties (Baumgart et al., 2007; Darfeuille-Michaud et al., 2004; Darfeuille-Michaud et al., 1998; Elliott et al., 2013).

Microbial dysbiosis has also been identified in CRC (Jobin, 2013), and *E. coli* seem to play an important role in the pathogenesis of this disease (Arthur and Jobin, 2013). For example, mono-association of the adherent-invasive *E. coli* (AIEC) mouse strain NC101, which produces colibactin, enhanced colonic tumor development in azoxymethane (AOM)-treated interleukin-10-knockout (*Il-10*^−/−^) mice (Table 1), a mouse model of colitis-associated CRC (Arthur et al., 2012). The human CRC mucosa-associated *E. coli* strain 11G5 promoted intestinal neoplastic changes when introduced in *Apc^min/^*^+^ mice, which carry a mutation in one allele of the tumor suppressor gene adenomatous polyposis coli (*Apc*) and are genetically predisposed to developing intestinal tumors (Bonnet et al., 2014) (Table 1). Another human CRC *E. coli* isolate, CCR20, increased tumor formation in AOM–dextran-sulphate-sodium (DSS)-treated mice (Cougnoux et al., 2014) (Table 1). These *E. coli* strains thus constitute a novel group of ‘alpha bugs’. Importantly, a high prevalence of mucosa-associated *E. coli*, especially those producing cyclomodulins (bacterial toxins and effectors that interfere with the eukaryotic cell cycle), is observed in individuals with CRC (Arthur et al., 2012; Bonnet et al., 2014; Buc et al., 2013; Martin et al., 2004; Prorok-Hamon et al., 2014). Therefore, AIEC represents a group of microorganisms that is implicated in the pathogenesis of both IBD and CRC. In the following section, we will discuss important host-bacteria and bacteria-bacteria interactions that modulate *E. coli* colonization and fitness in the intestine, and review key mechanisms by which *E. coli* mediate carcinogenesis.

**Table 1.**
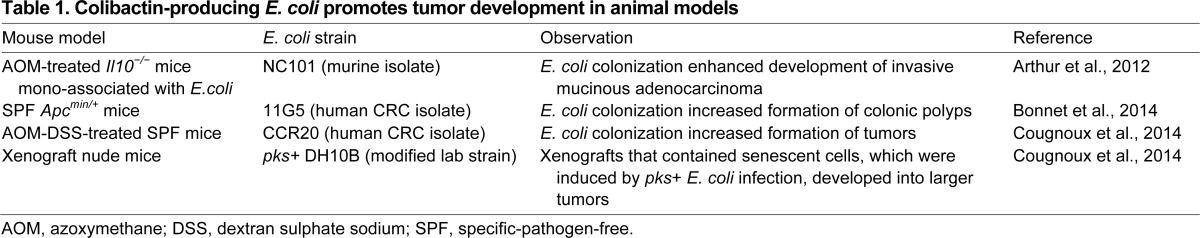
Colibactin-producing *E. coli* promotes tumor development in animal models

## Factors affecting *E. coli* colonization and fitness in the intestine

### Host regulation at gut homeostasis

*E. coli* colonize the gut of a human infant within hours of birth, largely owing to their ability to respire oxygen that is present in the newborn’s intestine (Palmer et al., 2007), and thereafter establishes a symbiotic relationship with their host.

**Fig. 1.**
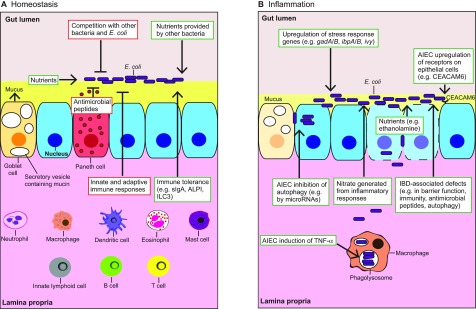
**Events modulating *E. coli* colonization and fitness in the intestine.** Events promoting *E. coli* fitness are illustrated in green boxes and those inhibiting *E. coli* growth are shown in red boxes. (A) At homeostasis, bacteria such as *E. coli* are prevented from attaching to epithelial cells (blue cells; for simplicity, villi are not shown) by a mucus layer present at the apical surface of the gut epithelium. The host immune system and the Paneth-cell-derived antimicrobial peptides regulate microbial growth. Major cell types that are involved in immune regulation of the gut microbes are illustrated in the lamina propria, where they are found. The host gut provides nutrients to support *E. coli* colonization, and immune tolerance mechanisms help to maintain a healthy *E. coli* community in the intestine. *E. coli* colonization is also modulated by bacterial competition for nutrients. Other bacteria can provide nutrients (e.g. mono- and disaccharides) to *E. coli* and promote its growth. (B) During intestinal inflammation, mucus depletion facilitates *E. coli* attachment to the epithelium. *E. coli* adjust their metabolism, and upregulate stress response genes to promote their survival. *E. coli* can take advantage of inflammation by using nutrients provided by dead cells and by respiring host-derived nitrate. Adherent invasive *E. coli* (AIEC) can disrupt the epithelial barrier, inhibit epithelial cell autophagy to promote intracellular survival, and upregulate surface receptors on epithelial cells to increase adherence. AIEC infection stimulates TNF-α expression in macrophages, which promotes their intracellular replication. IBD-associated deficiencies such as reduced secretion of antimicrobial peptides due to Paneth cell dysfunction (Paneth cells not shown) can further allow bacterial attachment, invasion and growth. CEACAM6, carcinoembryonic antigen-related cell adhesion molecule 6; *gadA/B*, glutamic acid decarboxylase A and B; ALPI, intestinal alkaline phosphatase; *ibpA/B*, inclusion body protein A and B; ILC3, group 3 innate lymphoid cells; *ivy*, inhibitor of C-type lysozyme; sIgA, secretory immunoglobulin A; TNF-α, tumor necrosis factor α.

The gut luminal surface is covered by a single layer of epithelial cells, which is composed of absorptive enterocytes, secretory enteroendocrine cells, mucus-producing goblet cells, Paneth cells and a stem cell compartment that assures renewal of all cell lineages. Underneath the gut epithelium is the lamina propria, which harbors diverse immune cell populations (neutrophils, macrophages, dendritic cells, innate lymphoid cells, B and T cells, etc.) that are involved in gut immunity (Fig. 1A). At gut homeostasis, uncontrolled interaction between *E. coli*, or other commensal bacteria, and intestinal epithelial cells is prevented by a layer of mucus on the apical side of the epithelium (Bergstrom et al., 2010; Johansson et al., 2008) (Fig. 1A). The organization of this protective mucus system varies along the intestine. Mucus in the small bowel is unattached to the gut epithelium and is easily removable, whereas mucus in the colon forms two layers: one inner dense and attached mucus layer and an outer loose and unattached layer (Johansson et al., 2013). Mucus in both the small and the large intestine is composed of the same mucin protein, MUC2 (mucin 2), but the distinct two-layer organization in the colon provides a stronger protective barrier against the bacteria that are abundantly present in this region of the intestine (Johansson et al., 2013). Interestingly, mucin was reported to facilitate biofilm formation by *E. coli* (Bollinger et al., 2003; Bollinger et al., 2006), suggesting its potential role in modulating *E. coli* colonization in the gut.

Commensal microorganisms, such as *E. coli*, are constantly monitored by the host immune system (Slack et al., 2009) (Fig. 1A). The innate immune receptors expressed by intestinal epithelial cells and mucosal immune cells can respond to various *E. coli*-derived antigens. For example, Toll-like receptor 4 (TLR4) responds to lipopolysaccharide (LPS); Toll-like receptor 2 (TLR2) and NOD2 respond to peptidoglycan; and Toll-like receptor 5 (TLR5) responds to flagellin. These innate immune responses result in activation and recruitment of phagocytic cells, such as neutrophils and macrophages, which eliminate the microbes that breach the mucosal barrier. The adaptive immune system, which consists mainly of highly specialized B and T cells, provides additional protection against invading microbes and keeps the microbial population in check (Kamada et al., 2013). In particular, intestinal B cells produce large amounts of non-inflammatory immunoglobulin A (IgA) antibodies, which play important roles in maintaining appropriate bacterial communities within specific intestinal segments (Cerutti and Rescigno, 2008). Interestingly, similarly to mucin, IgA can also promote *E. coli* biofilm formation (Bollinger et al., 2003; Bollinger et al., 2006), and potentially modulates *E. coli* colonization and persistency in the intestine (Barbas et al., 2009). Another layer of protection is provided by Paneth cells, a type of cell found in the epithelium of the small intestine and appendix, which secrete a consortium of antimicrobial peptides. Many of these antimicrobial peptides are active against *E. coli* (Cunliffe and Mahida, 2004) and might restrict *E. coli* colonization and growth in the intestine (Fig. 1A).

Importantly, the host has also evolved immunoregulatory mechanisms that prevent excessive immune activation in response to symbiotic microbes, such as commensal *E. coli* (Fig. 1A). For example, the Gram-negative bacterium-derived LPS, upon activation of the host TLR4, upregulates intestinal alkaline phosphatase (ALPI), which functions to dephosphorylate LPS and thereby dampen the LPS-TLR4 innate immune response (Bates et al., 2007). In addition, secretory IgA in the gut mucosa can downregulate the expression of pro-inflammatory bacterial epitopes by commensal bacteria, and prevent microbes and microbial components from attaching to the gut epithelium (Cerutti and Rescigno, 2008). More recently, group 3 innate lymphoid cells (ILC3) have been demonstrated to inhibit inflammatory T-cell responses to commensal bacteria in the intestine (Hepworth et al., 2013). A delicate balance between immune surveillance and immune tolerance is required to maintain the mutually beneficial relationship between the host and the gut microbiota.

It should be emphasized that here we focus on commensal bacterial strains, not highly virulent strains (enteropathogenic *E. coli*, enterohemorrhagic *E. coli*, etc.) that have evolved sophisticated machineries to subvert the aforementioned regulatory mechanisms (Clements et al., 2012).

### Bacterial competition

The intestine provides a nutrient-rich environment for commensal microbes such as *E. coli*. Upon intestinal colonization, *E. coli* genes involved in carbohydrate and amino acid metabolism and transport are strongly induced (Alpert et al., 2009). Similar metabolic function changes are observed in other commensal bacteria, for example, in *Bifidobacterium longum* (Yuan et al., 2008) and *Lactococcus lactis* (Roy et al., 2008). The high number of microbes that colonize the intestine implies that competition for common nutrients potentially exists among different commensal bacterial species, which could restrict the growth of *E. coli* in the intestine (Fig. 1A). The bioavailability of certain substrates could be critical for *E. coli* fitness in the intestine given that impaired carbohydrate metabolism or deficient purine and pyrimidine synthesis significantly diminishes the ability of *E. coli* to colonize the mouse intestine (Chang et al., 2004; Vogel-Scheel et al., 2010).

However, the relationship between commensal bacteria, in terms of nutritional needs, is not always competitive. Commensal *E. coli* strains colonize the mouse large intestine by growing in intestinal mucus (Miranda et al., 2004; Myhal et al., 1982; Sweeney et al., 1996a; Sweeney et al., 1996b; Wadolkowski et al., 1988) (Fig. 1A). Mucus is an important source of carbohydrates, mostly in the form of polysaccharides, which support bacterial colonization and growth (Fabich et al., 2008). However, *E. coli* do not secrete extracellular polysaccharide hydrolases (Henrissat and Davies, 1997; Hoskins et al., 1985) and therefore cannot directly use mucin-derived polysaccharides as a carbohydrate source. It is likely that polysaccharide-degrading anaerobes provide the mono- and disaccharides that *E. coli* need for growth (Conway et al., 2004), thereby promoting *E. coli* fitness in the intestine (Fig. 1A). The model of anaerobes feeding *E. coli* has been described by Leatham-Jensen et al. as the ‘restaurant’ hypothesis (Leatham-Jensen et al., 2012), which states that *E. coli* inhabits mixed biofilms in the intestine and grows on sugars served locally by surrounding anaerobes.

Colonization competition could also occur within the same species. For example, germ-free mice that are colonized with *B. fragilis* are resistant to further colonization of the same species (Lee et al., 2013). This ‘colonization resistance’ phenomenon could arise as a result of competition for a limited-space niche, as in the case of *B. fragilis* (Lee et al., 2013), or because of competition for nutrients, as has been proposed for *E. coli* (Fabich et al., 2008; Maltby et al., 2013) (Fig. 1A). Carbohydrates are fundamental nutrients for *E. coli*, and different *E. coli* strains can compete for the same kinds of sugar molecule, affecting each other’s fitness (Fabich et al., 2008; Maltby et al., 2013). It is not known whether different *E. coli* strains also compete for a specific colonization niche in the intestine, like *B. fragilis*.

Moreover, quorum sensing, a system that bacteria use to coordinate gene expression and behavior according to their local population density, could also be involved in modulating *E. coli* colonization and dissemination along the intestine. The quorum-sensing transcriptional regulator SdiA has been found to be necessary for *E. coli* O157:H7 colonization of the bovine intestine (Sharma and Bearson, 2013). Additionally, a mutant strain of *E. coli* O157:H7 that lacks the *qseBC*-encoded quorum-sensing system, which regulates the motility of *E. coli* O157:H7 in response to bacterial autoinducer 3 and the mammalian stress hormones epinephrine and norepinephrine, outcompetes its parental strain during colonization of the bovine intestine (Sharma and Casey, 2014). However, it remains unknown whether such mechanisms are involved in maintaining the colonization niche and growth of commensal *E. coli* in the human intestine.

### Intestinal inflammation

**Fig. 2.**
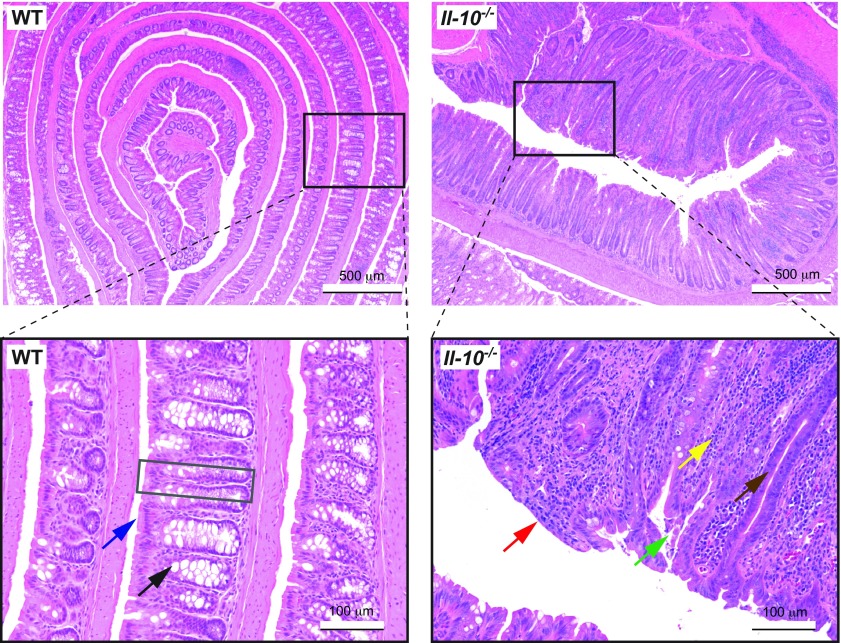
**Intestinal inflammation creates a different environment for the gut microbes.** Hematoxylin and eosin (H&E)-stained colon Swiss roll sections from a 9-month-old wild-type (WT) mouse (left; healthy) and an age-matched interleukin-10 knockout (*Il-10*^−/−^) mouse (right, inflamed). (Left, inset) The WT mouse colon has a well-organized lining of villi (gray box), with the presence of columnar epithelial cells (with microvilli; blue arrow) and goblet cells (black arrow). (Right, inset) The inflamed *Il-10*^−/−^ mouse colon shows loss of the regular villus structures, thickening of the mucosa, crypt elongation (brown arrow), infiltration of inflammatory immune cells (yellow arrow), depletion of goblet cells, disruption of the brush border (red arrow) and loss of surface epithelial cells (green arrow).

Intestinal inflammation creates a markedly different environment for gut microbes. For example, the healthy intestine has a well-organized structure, and can be recognized by the lining of regular villi and the presence of columnar epithelial cells and goblet cells (Fig. 2). In comparison, the inflamed gut as seen in the *Il-10*^−/−^ mouse, a spontaneous mouse model of colitis triggered by microbial colonization (Sellon et al., 1998), often shows loss of the villus structures, thickening of the mucosa, infiltration of inflammatory immune cells, depletion of goblet cells, disruption of the brush border and loss of surface epithelial cells (Fig. 2).

As highly versatile microorganisms, *E. coli* can quickly adapt to the new environment. *E. coli* adjust their metabolic functions and upregulate stress response genes [e.g. glutamic acid decarboxylase A and B (*gadA* and *gadB*), inclusion body protein A and B (*ibpA* and *ibpB*) and inhibitor of C-type lysozyme (*ivy*)] during intestinal inflammation (Fig. 1B), as shown in experimental models (Patwa et al., 2011; Schumann et al., 2012). Inflammation is characterized by oxidative stress (high levels of reactive oxygen and nitrogen species), which is hazardous to bacteria. However, *E. coli* are equipped with a variety of responding strategies to help their survival (Imlay, 2013). It is worth noting that different *E. coli* strains likely rely on distinct mechanisms to survive in the inflamed environment. For example, as compared with *E. coli* NC101, the *E. coli* strain Nissle 1917 shows a much stronger upregulation of *ivy* expression, which promotes cell resistance to lysis by lysozyme, when colonizing the gut of germ-free *Il-10*^−/−^ mice (Schumann et al., 2013).

*E. coli* might even exploit the environment in an inflamed gut to obtain a growth advantage. Chronic intestinal inflammation results in continuous epithelial cell death and tissue damage. The dead cells that slough off the intestinal luminal surface could provide extra nutrients, such as ethanolamine, to support *E. coli* growth (Bertin et al., 2011; Garsin, 2010) (Fig. 1B). The disruption of gut epithelial barrier during inflammation exposes deep gut tissue for *E. coli* translocation and infection (Fig. 1B). Recently, it was demonstrated that nitrate, a by-product of the host inflammatory response, can be used by *E. coli* for anaerobic respiration, thus conferring benefits to *E. coli* in the inflamed gut (Spees et al., 2013; Winter et al., 2013) (Fig. 1B). Although this represents a mechanism that potentially helps *E. coli* to outcompete fermenting anaerobes during intestinal inflammation, other IBD-associated factors could also promote bacterial growth. For example, individuals with IBD often have impaired innate and adaptive immune responses, with mutations in *NOD2*, *NF-ΚB1* (nuclear factor kappa-light-chain-enhancer of activated B cells 1) and interleukin-related genes (*IL2*, *IL21*, *IL23R*, etc.) (Jostins et al., 2012), which might further facilitate *E. coli* adherence and invasion (Fig. 1B). In addition, defects in Paneth cell function in individuals with IBD (Wehkamp et al., 2005) and subsequently decreased secretion of antimicrobial peptides (Salzman et al., 2010) (Fig. 1B) could impair the control of bacterial infection by the intestinal mucosa, giving rise to a higher prevalence of *E. coli*.

*E. coli*, especially AIEC (Boudeau et al., 1999), have been extensively studied in individuals with IBD. AIEC type 1 pili and flagellae bind to the epithelial cell surface protein CEACAM6 (carcinoembryonic antigen-related cell adhesion molecule 6), which is upregulated in individuals with Crohn’s disease (Barnich et al., 2003; Barnich et al., 2007; Boudeau et al., 2001). Overexpression of CEACAM6 promotes AIEC colonization, and AIEC infection can further upregulate CEACAM6 (Fig. 1B) through the induction of the pro-inflammatory cytokines interferon γ (IFN-γ) and TNF-α (Barnich et al., 2007). The AIEC outer-membrane protein OmpA interacts with the endoplasmic reticulum (ER) stress-response glycoprotein Gp96, which is also overexpressed at the apical surface of ileal epithelial cells in individuals with Crohn’s disease (Rolhion et al., 2010). ER stress is commonly associated with inflammation (Garg et al., 2012; Kaser et al., 2013), and AIEC might take advantage of the ER stress response that occurs in individuals with IBD to increase its adherence to the intestinal epithelia. Moreover, the AIEC strain LF82, which was isolated from an IBD patient, has been shown to disrupt the polarized gut epithelial barrier and to replicate inside epithelial cells (Wine et al., 2009), implicating AIEC in the mucosal-barrier dysfunction observed in individuals with IBD (D’Incà et al., 2006; Hilsden et al., 1999; Wyatt et al., 1993).

A distinct feature of AIEC is the ability to survive and replicate in phagolysosomes within macrophages (De la Fuente et al., 2014; Glasser et al., 2001). AIEC infection stimulates expression of *TNF-α* in macrophages (Glasser et al., 2001). Interestingly, exogenous TNF-α treatment results in dose-dependent increases in the number of LF82 inside macrophages, whereas neutralization of TNF-α secreted by infected macrophages significantly reduces the number of intramacrophagic bacteria (Bringer et al., 2012). Therefore, AIEC-infection-induced TNF-α could promote bacterial growth inside macrophages (Fig. 1B).

Individuals with IBD who have mutations in innate response genes [e.g. *NOD2*, *ATG16L1* (autophagy related 16-like 1), *IRGM* (immunity-related GTPase family M)] might also have impaired autophagic responses (Jostins et al., 2012), which could contribute to the overgrowth of AIEC, given that autophagy restricts the replication of intracellular AIEC (Lapaquette et al., 2010; Lu et al., 2014) (Fig. 1B). In fact, the AIEC strain LF82 has been shown to attenuate host-mediated autophagy by inducing microRNAs 30C and 130A, which downregulate genes required for the autophagic response (Nguyen et al., 2014) (Fig. 1B). A proper autophagy response is essential for intestinal homeostasis: defective function of ATG16L1 or XBP1 (X-box binding protein-1), which are involved in autophagy, in Paneth cells promotes inflammation in the ileum in mouse models (Adolph et al., 2013). Whether these mice are impaired in handling any microorganism or a specific one (e.g. *E. coli*) is currently unknown.

In summary, at gut homeostasis, *E. coli* are tightly controlled by a variety of host- and bacteria-derived mechanisms. During intestinal inflammation, they can quickly adjust to and even exploit the inflamed environment to gain a growth advantage. In the following section, we will review recent studies that demonstrate the cancer-promoting role of *E. coli* and discuss the key mechanisms by which dysregulated *E. coli* could mediate intestinal carcinogenesis.

## *E. coli* cancer-promoting mechanisms

**Fig. 3.**
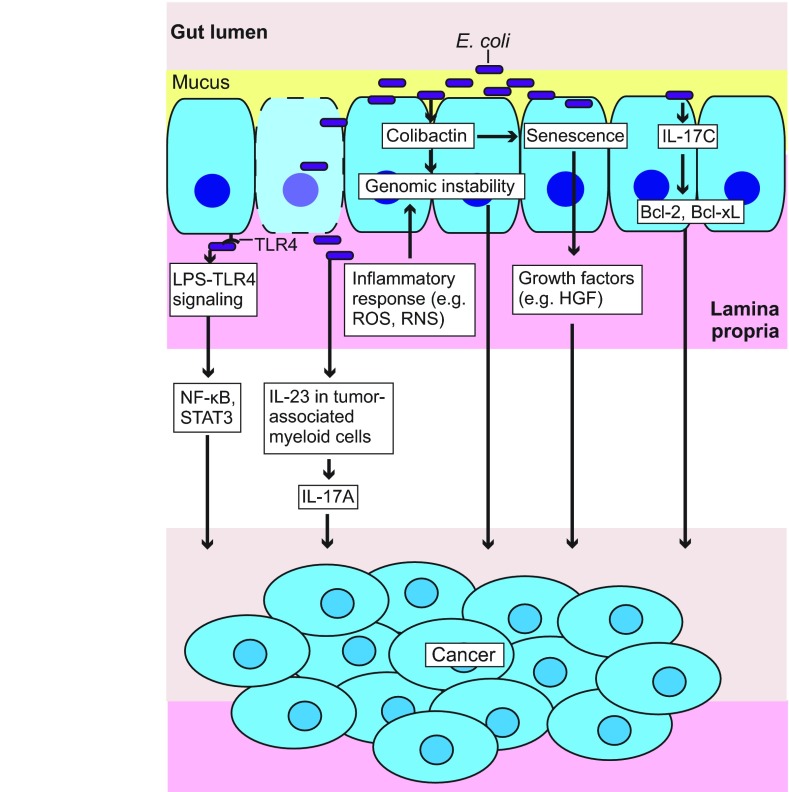
***E. coli* cancer-promoting mechanisms.** *E. coli*-induced innate immune responses (e.g. LPS-TLR4 signaling) can result in enhanced cell proliferation and survival through activating NF-κB and STAT3. Bacterial products (e.g. LPS) can promote cell proliferation by activating myeloid-cell-derived IL-23 and subsequently IL-17A. *E. coli* can induce epithelial-cell-derived IL-17C and subsequently Bcl-2 and Bcl-xL to enhance cell survival. Reactive oxygen and nitrogen species (ROS, RNS) produced by innate immune cells (e.g. macrophages and neutrophils) during *E. coli* infection, and *E. coli*-derived genotoxins (e.g. colibactin) can cause DNA damage and thus genomic instability. Colibactin-producing *E. coli* infection causes epithelial cell senescence, and senescent cells release growth factors (e.g. HGF) to promote cell proliferation. Bcl-2, B-cell lymphoma 2; Bcl-xL, B-cell lymphoma-extra large; HGF, hepatocyte growth factor; IL-17A, interleukin 17A; IL-17C, interleukin 17C; IL-23, interleukin 23; LPS, lipopolysaccharide; NF-κB (nuclear factor kappa-light-chain-enhancer of activated B cells; ROS, reactive oxygen species; RNS, reactive nitrogen species; STAT3, signal transducer and activator of transcription 3; TLR4, Toll-like receptor 4.

*E. coli* that colonize in the gut provide a source of antigens and toxins that foster host inflammatory responses, many of which have been linked to carcinogenesis (discussed earlier). For example, microbial activation of the innate immune pathways, in particular the LPS-TLR4 signaling pathway, can promote cancer development (Schwabe and Jobin, 2013). TLR4 is overexpressed in individuals with colitis-associated CRC (Fukata et al., 2007), and constitutive activation of TLR4 enhances cancer development in mouse models of this disease (Fukata et al., 2011). Therefore, microbial LPS could be involved in CRC development by activating TLR4 (Fig. 3). Although the cancer-promoting effect of LPS-TLR4 signaling is probably negligible at gut homeostasis, it could be markedly increased in IBD, where Gram-negative bacteria such as *E. coli* are abundantly present and the host gut barrier function and immune system are impaired.

A key cancer-promoting downstream effect of innate immune signaling could be the induction of cell proliferation and survival pathways mediated by NF-κB and STAT3 (signal transducer and activator of transcription 3) (Elinav et al., 2013; Schwabe and Jobin, 2013) (Fig. 3). It should be emphasized though that innate immune responses have complex functions, and some innate immune sensors, such as NOD-like receptors NOD1, NOD2, NLRP3 and NLRP6, have been shown to play cancer-suppressing roles (Elinav et al., 2013; Schwabe and Jobin, 2013). Another model of inflammation-mediated carcinogenesis involves gut infiltration of phagocytes and their generation of reactive species, which causes DNA damage and thus genomic instability (Mangerich et al., 2012) (Fig. 3). Enhanced cell proliferation and survival combined with increased genomic instability heightens the risk of carcinogenesis.

Using the CPC-APC mouse model (mice that have *Apc* allelic loss specifically in the colonic epithelium and develop tumors primarily in the distal colon), Grivennikov et al. recently showed that microbial products, such as LPS, could activate interleukin 23 (IL-23) in tumor-associated myeloid cells and, subsequently, interleukin 17A (IL-17A) to drive tumor growth and progression (Grivennikov et al., 2012). This IL-23–IL-17A adaptive immune response might also contribute to the cancer-promoting effects of *E. coli* (Fig. 3). It is worth noting that the pro-tumorigenic function of LPS requires the presence of microbes, because LPS administration reduces colonic carcinogenesis in germ-free AOM-DSS-treated mice (Zhan et al., 2013).

More recently, Song et al. have shown that IL-17C, which is induced in intestinal epithelial cells by the gut microbiota, upregulates the anti-apoptotic factors Bcl-2 (B-cell lymphoma 2) and Bcl-xL (B-cell lymphoma-extra large), resulting in enhanced epithelial cell survival and thereby carcinogenesis (Song et al., 2014). Noticeably, *E. coli* colonization upregulates IL-17C in the colon of DSS-treated germ-free or antibiotic-treated mice (Song et al., 2014). Thus, the induction of IL-17C in intestinal epithelial cells could represent another mechanism by which *E. coli* promotes cancer development (Fig. 3).

Genomic instability can also arise from DNA damage induced by *E. coli*-derived genotoxins such as cytolethal distending toxin (CDT) and colibactin. CDT is the best-studied bacterial genotoxin and is secreted by many Gram-negative bacterial species, including *E. coli* (Guerra et al., 2011). CDT consists of two binding subunits (CdtA and CdtC), which mediate CDT internalization into target cells, and a catalytic subunit (CdtB), which cleaves DNA and induces DNA double-strand breaks (DSBs) (Guerra et al., 2011). The genotoxic property of CDT could be important for the carcinogenic potential of CDT-producing bacteria, because CdtB-mutant strains of *Campylobacter jejuni* (Fox et al., 2004) and of *Helicobacter cinaedi* (Shen et al., 2009) fail to elicit intestinal hyperplasia in *NF-κB*-deficient mice and intestinal dysplasia in *Il-10*^−/−^ mice, respectively. However, it is not clear to what extent CDT contributes to the pro-tumorigenic potential of *E. coli*.

The genotoxin colibactin, which is encoded by a ~54-kb polyketide synthase (*pks*) pathogenicity island, is predominantly found in *E. coli* of the phylogroup B2 (Nougayrède et al., 2006). Approximately 75% of *E. coli* strains within the phylogroup B2 are *pks*-positive (*pks*+) (Johnson et al., 2008). Other bacterial species, such as *Proteus mirabilis* and *Klebsiella pneumoniae*, have also been found to produce colibactin (Putze et al., 2009). Colibactin-producing *E. coli* induce DSBs *in vitro* and *in vivo*, in various cells, including intestinal epithelial cells (Arthur et al., 2012; Cuevas-Ramos et al., 2010; Nougayrède et al., 2006; Secher et al., 2013). Unlike CDT, the genotoxic activity of colibactin requires cell-bacterium contact (Nougayrède et al., 2006). To date, little is known about how colibactin exerts its genotoxic effect, although the induction of DSBs is a key feature of its known activity. From the view point of microbial fitness, the presence of colibactin seems to correlate with the capacity of *E. coli* to maintain long-term colonization in the intestine (Nowrouzian and Oswald, 2012). The mechanism by which colibactin promotes colonization persistence in the intestine remains to be elucidated.

Several recent studies have demonstrated the cancer-promoting effect of *E. coli* and revealed that colibactin is a major contributing factor to *E. coli*-mediated intestinal carcinogenesis (Fig. 3). Arthur et al. reported that intestinal colonization of *Il-10*^−/−^ mice by the human commensal *Enterococcus faecalis* strain OG1RF or the colibactin-producing AIEC strain NC101 caused comparable intestinal inflammation, but only NC101 was able to induce cancer in AOM-treated *Il-10*^−/−^ mice (Arthur et al., 2012) (Table 1). This study also shows that colibactin is required for *E. coli* to mediate carcinogenesis in these mice, because a mutant strain of NC101 that lacks the *pks* pathogenicity island failed to elicit the carcinogenic effect even though intestinal inflammation was still severe (Arthur et al., 2012). This indicates that inflammation alone is not sufficient for CRC development, and provides the first evidence that specific bacterial activities, such as the production of colibactin by *E. coli*, are required for intestinal carcinogenesis. It is worth noting that this study uses *Il-10*^−/−^ mice mono-associated with *E. coli* or *E. faecalis* (Arthur et al., 2012), and therefore the contribution of colibactin-producing *E. coli* to inflammation-related CRC pathogenesis in the presence of a complex microbiota remains to be elucidated. Considering the murine origin of *E. coli* NC101, it would also be interesting to test other *E. coli* strains, for example, clinical human CRC isolates, in this model.

Importantly, *E. coli* that produce colibactin show a higher prevalence in human cases of IBD and CRC than in non-IBD non-CRC controls (Arthur et al., 2012; Bonnet et al., 2014; Buc et al., 2013; Martin et al., 2004; Prorok-Hamon et al., 2014). These findings suggest that *E. coli* prevail during intestinal inflammation and contribute to carcinogenesis by producing colibactin.

The key role of *E. coli* colibactin in CRC development was subsequently confirmed in the *Apc^min^*^/+^ mouse model (Bonnet et al., 2014). The human CRC-associated *E. coli* strain 11G5, which also produces colibactin, was shown to stimulate the development of colonic polyps in specific-pathogen-free (SPF) *Apc^min^*^/+^ mice (Bonnet et al., 2014) (Table 1). In contrast, the *E. coli* strain K-12 MG1655, which does not produce colibactin, showed no pro-tumorigenic effect in this model (Bonnet et al., 2014). This study demonstrates the carcinogenic potential of colibactin-producing *E. coli* in the non-inflamed gut in the presence of a complex microbiota. This study also indicates that genetic predisposition (in this case, altered APC signaling) is required for *E. coli* to elicit its carcinogenic effect because wild-type mice infected with *E. coli* 11G5 did not show any neoplastic changes (Bonnet et al., 2014). Noticeably, *Apc^min/+^* mice colonized by *E. coli* 11G5 did not develop advanced carcinomas (Bonnet et al., 2014), suggesting that additional factors are required for the full development of cancers in this model. It is also worth noting that this *E. coli*/*Apc^min/+^* model involves pre-treating mice with streptomycin before *E. coli* infection (Bonnet et al., 2014). Because this broad-spectrum antibiotic can drastically perturb the commensal microbiota, this experimental procedure precludes investigation of the role for an intact commensal microbial community in regulating *E. coli* colonization and *E. coli*-mediated carcinogenesis.

Colibactin-producing *E. coli* have also been found to induce cell senescence *in vitro*, a phenomenon that might be linked to the carcinogenic effect of the bacteria (Cougnoux et al., 2014; Secher et al., 2013). Recently, Cougnoux et al. reported that senescent cells arising from a colibactin-producing *E. coli* infection secreted growth factors and promoted tumor growth in mouse models (Cougnoux et al., 2014) (Fig. 3). Nude mice (genetically engineered mice that have a greatly reduced number of T cells and therefore mount no rejection response to implanted materials) injected with a mixture of senescent HCT116 cells, induced by *pks+ E. coli* infection, and uninfected cells developed larger xenograft tumors than did mice injected with non-infected cells alone (Cougnoux et al., 2014) (Table 1). This enhanced tumor-growth phenotype was abrogated by the administration of HGF (hepatocyte growth factor) inhibitor, suggesting that the pro-tumorigenic effect of *pks+ E. coli*-induced senescent cells is mainly mediated by HGF (Cougnoux et al., 2014). In addition, the authors found that the human *pks+ E. coli* strain CCR20, but not the isogenic CCR20 *pks* mutant, significantly increased the number of tumors in AOM-DSS-treated mice (Cougnoux et al., 2014) (Table 1). Interestingly, CCR20 colonization did not affect inflammation, neoplastic grade or tumor size in this mouse model (Cougnoux et al., 2014). This study indicates that colibactin-producing *E. coli* mediate carcinogenesis through a variety of mechanisms, including senescence-induced HGF production and induction of host genomic instability (Fig. 3). However, given that, in the AOM-DSS mouse model, microbial colonization of germ-free mice attenuated colonic polyp formation (Zhan et al., 2013), this model might not be ideal for studying the contribution of *pks+ E. coli* to CRC. Moreover, this model requires the use of streptomycin (Cougnoux et al., 2014), as previously mentioned, which could have confounding effects on the interplay between the host and the microbiota.

In addition to genotoxin-induced DNA damage, *E. coli* might also encode factors, such as MutY (Khan and Cash, 2013), that could interfere with host-cell DNA-repair mechanisms to further increase genomic instability in the host and promote cancer susceptibility. Recently, Maddocks et al. reported that enteropathogenic *E. coli*-secreted effector protein EspF was required for the depletion of host-cell DNA mismatch repair (MMR) proteins (Maddocks et al., 2013). Future research will need to identify other *E. coli*-associated factors that interfere with host DNA repair, characterize their presence and working mechanisms in various *E. coli* strains, and evaluate their clinical relevance.

Other *E. coli* virulence factors, such as cytotoxic necrotizing factor 1 (CNF1) (Fabbri et al., 2013), have also been proposed to contribute to the cancer-promoting potential of *E. coli*. However, the *in vivo* relevance of these bacterial factors to human CRC remains to be determined.

## Conclusions and future perspectives

*E. coli* is a common gut commensal, the colonization of which is tightly controlled in healthy individuals by both host- and bacteria-derived mechanisms. However, overrepresentation of *E. coli* is often observed in disease states, for example, in IBD and CRC. As an extremely versatile species, *E. coli* is able to adapt to and even take advantage of the environment present in the inflamed intestine to gain a growth advantage.

In the last decade, substantial evidence has been obtained from both clinical and basic research that the gut microbiota can profoundly affect intestinal inflammation and tumor development. In particular, certain members of the microbiota (so-called ‘alpha bugs’, which possess unique virulence traits) have been identified as direct drivers of intestinal carcinogenesis. The first example of such carcinogenic bacteria was the enterotoxigenic *B. fragilis* (Wu et al., 2009). However, although the microorganism has been linked to inflammatory diarrhea, its involvement in human IBD and CRC remains unclear. The recently reported colibactin-producing *E. coli* might be part of this novel group of ‘alpha bugs’.

Several research groups have recently demonstrated the carcinogenic role of colibactin-producing *E. coli* by using mouse models (Arthur et al., 2012; Bonnet et al., 2014; Cougnoux et al., 2014). However, certain limitations associated with the experimental models used in these studies should be recognized (discussed earlier). Using a model of vertical bacterial transmission (from mother to pup) of AIEC would help to determine the physiological role of early colonization by these bacteria in intestinal homeostasis and pathology. Such a model has recently been used to demonstrate that *pks+ E. coli* colonization results in DNA damage in neonates and genotoxic stress in adult mice (Payros et al., 2014).

Recent advances in our understanding of the regulation and function of *E. coli* in gut pathologies, such as IBD and CRC, also raise important new questions. It would be particularly interesting to know, for example, what factors and mechanisms lead to the dysbiotic state in the inflamed gut and especially to the high prevalence of *E. coli*. Because the microbiota responds to anti-inflammatory treatment (Rooks et al., 2014), it would be important to determine whether the activity of genotoxic factors, such as colibactin, is affected by the intestinal environment and therapeutic interventions. We recently observed transcriptional changes in *pks*-associated genes in *E. coli* strain NC101 during the development of colitis-associated CRC (Arthur et al., 2014), suggesting a dynamic microbial response to the intestinal environment. Colibactin modulates numerous aspects of host responses (DSB, cell senescence and transformation), and we need more information on how the microbial product is assembled, regulated and transported to mediate host responses. Purifying colibactin and resolving its structure would help to reveal the functional mechanisms underlying its genotoxic activity. This knowledge would be particularly valuable for designing drugs that could interfere with the activity of colibactin and prevent carcinogenesis mediated by colibactin-producing *E. coli*. Another interesting question is how other cyclomodulins (such as CDT) and toxins (such as CNF1) contribute to the pro-tumorigenic potential of *E. coli*.

IBD and CRC are likely polymicrobially driven, and dissecting the microbial-community actions that foster dysbiosis and CRC development is essential for understanding the disease etiology and for developing future therapies. Pathogenic traits of some bacteria might necessitate interaction with other members of the microbial community. Understanding the ‘cooperation’ between disease-associated bacteria is crucial for defining the role of the gut microbiota in IBD and CRC.

It is likely that microbial production of metabolites (e.g. SCFA), toxins (e.g. colibactin) and gas (e.g. H_2_S) influences the course of disease development, with some factors preventing while others promote it. The biological effect of a metabolite might depend on other environmental factors (diet, inflammation, stress, etc.) as well as host genetics. For example, a fiber-rich diet fosters the production of butyrate and attenuates CRC development through the enhanced function of regulatory T cells (Treg) (Singh et al., 2014). However, butyrate production induced by a carbohydrate-rich diet promotes neoplastic development in the intestine of *Apc^min/+^* mice that are also deficient for the DNA mismatch repair gene MutS homolog 2 (*Msh2*) (Belcheva et al., 2014). Dissecting the contribution of the microbial metabolites and their relationship with host genetic factors and other environmental components would provide a clearer picture of the events by which microbes influence intestinal pathology.

Although deciphering the function and activities of gut microbes that are associated with intestinal protection and pathology remains a daunting task, one that requires a wide range of expertise (microbiology, genomics, proteomics, metabolomics, etc.), it is evident that this field of research holds much promise for disease prevention and treatment.
